# Intravenous Sphingosylphosphorylcholine Protects Ischemic and Postischemic Myocardial Tissue in a Mouse Model of Myocardial Ischemia/Reperfusion Injury

**DOI:** 10.1155/2010/425191

**Published:** 2011-01-03

**Authors:** Christine Herzog, Martina Schmitz, Bodo Levkau, Ilka Herrgott, Jan Mersmann, Jan Larmann, Kai Johanning, Michael Winterhalter, Jerold Chun, Frank Ulrich Müller, Frank Echtermeyer, Reinhard Hildebrand, Gregor Theilmeier

**Affiliations:** ^1^Department of Anaesthesiology and Intensive Care Medicine, Hannover Medical School, 30625 Hannover, Germany; ^2^Institute for Anatomy and IZKF, Münster University Hospital, University of Münster, 48149 Münster, Germany; ^3^Institute of Pathophysiology, Center of Internal Medicine, University Hospital of Essen, 45122 Essen, Germany; ^4^Department of Anaesthesiology and IZKF, Münster University Hospital University of Münster, 48129 Münster, Germany; ^5^Department of Molecular Biology, Helen L. Dorris Child and Adolescent Neuropsychiatric Disorder Institute, The Scripps Research Institute, La Jolla, CA 92037, USA; ^6^Institute of Pharmacology and Toxicology, University of Münster, 48149 Münster, Germany

## Abstract

HDL, through sphingosine-1-phosphate (S1P), exerts direct cardioprotective effects on ischemic myocardium. It remains unclear whether other HDL-associated sphingophospholipids have similar effects. We therefore examined if HDL-associated sphingosylphosphorylcholine (SPC) reduces infarct size in a mouse model of transient myocardial ischemia/reperfusion. Intravenously administered SPC dose-dependently reduced infarct size after 30 minutes of myocardial ischemia and 24 hours reperfusion compared to controls. Infarct size was also reduced by postischemic, therapeutical administration of SPC. Immunohistochemistry revealed reduced polymorphonuclear neutrophil recruitment to the infarcted area after SPC treatment, and apoptosis was attenuated as measured by TUNEL. *In vitro*, SPC inhibited leukocyte adhesion to TNF*α*-activated endothelial cells and protected rat neonatal cardiomyocytes from apoptosis. S1P_3_ was identified as the lysophospholipid receptor mediating the cardioprotection by SPC, since its effect was completely absent in S1P_3_-deficient mice. We conclude that HDL-associated SPC directly protects against myocardial reperfusion injury *in vivo* via the S1P_3_ receptor.

## 1. Introduction

High-density lipoproteins (HDL) exert beneficial effects on cardiovascular pathologies not only due to their effects on reverse cholesterol transport, but in addition through pleiotropic effects on vessel wall biology [1]. In addition to its effects on vessel wall pathology, HDL has been shown to protect from myocardial injury and necrosis during reperfusion after ischemia [2]. Adhesion of leukocytes to the vascular endothelium and subsequent transmigration are a characteristic feature of inflammation. Reduced recruitment of leukocytes during reperfusion after ischemic insult has been shown to be beneficial in numerous experimental settings [3, 4]. Likewise, apoptotic cell death is a mainstay of tissue damage secondary to reperfusion injury after transient ischemia [5]. Antiapoptotic effects have been demonstrated to reduce reperfusion-induced tissue damage [6]. However, there is an ongoing debate as to the causal role of apoptosis in infarct enlargement during reperfusion injury. 

We have recently demonstrated that high-density lipoproteins (HDL) protect from myocardial damage during reperfusion after ischemia due to the anti-inflammatory and antiapoptotic effects of its sphingophospholipid (SPL) component, sphingosine-1-phosphate [7]. Like S1P, sphingosylphosphorylcholine (SPC) represents a major SPL species circulating with HDL. Several groups have shown that SPC, similar to S1P, has an inhibitory effect on TNF-*α*-induced expression of cell adhesion molecules in endothelial cells [8, 9]. SPL traveling with HDL have been shown to induce vasodilatation in contracted vessels [2, 10, 11]. There is, however, evidence for differential and even opposite effects when comparing S1P and SPC with respect to their effects in the cardiovascular system: S1P is a high-affinity ligand for the S1P-receptor family while SPC requires much higher concentrations to activate these G-protein-coupled receptors, which will activate NOS through Akt-phosphorylation in both cases. In addition, there is evidence for additional intracellular receptors or direct effectors of SPC and S1P. Engagement of these different receptors could indeed be a source for adverse effects of SPC compared to S1P. 

We, therefore, tested the hypotheses that (i) SPC—like S1P—exerts cardioprotective effects in an *in vivo *mouse model of myocardial ischemia with reperfusion and (ii) that such cardioprotective effects of SPC—if detectable—are also mediated via S1P_3_ receptors to ultimately result in reduced neutrophil recruitment and cardiomyocyte apoptosis to afford protection from postischemic myocardial necrosis.

## 2. Material and Methods

### 2.1. Materials

SPC and S1P (Sigma, Taufkirchen, Germany) from methanol stock solutions were air dried and dissolved in phosphate-buffered saline/1% bovine serum albumin and administered intravenously in 100 *μ*l/10 g body weight doses.

### 2.2. Myocardial Ischemia/Reperfusion

To assure strain independent effects of SPC treatment we used S1P_3_-deficient mice on a C57BL/6-background as well as an outbred Swiss strain [12]. Animals were strain matched, age matched, and sex matched and therefore used in a nonrandomized study design. Myocardial ischemia was induced with the approval of the Institutional Review Board and in accordance with the *Guide for the Care and Use of Laboratory Animals* published by the US National Institutes of Health as previously published [7]. Briefly, thoracotomy and ligation of the left anterior descending coronary artery (LAD) at the level of the left atrium were performed with silk-7-0 suture over a PE10-tubing in barbiturate-anesthetized mice for 30 minutes. The chest was closed before the animals were weaned from the ventilator and extubated. After 24 hours of reperfusion, animals were reanesthetized and perfused with 0.9% saline through the abdominal aorta. The coronary ligation was retied. 2% coomassie blue solution was injected to delineate the area at risk. The heart was sectioned into 5 equal slices from the apex to the base and immersed in 2-, 3-, 5-triphenyltetrazolium chloride (TTC) solution at 37°C. TTC development lasted 10 minutes before the sections were scanned, processed, and morphometrically analyzed for left ventricular area, area at risk, and area of infarction using Image J (NIH, Bethesda). Data are presented as the average percent infarct size per area at risk. SPC (0.625, 1.25, and 2.5 *μ*g/g body weight) was administered either 30 minutes before transient coronary ligation or therapeutically after myocardial ischemia with reinstitution of reperfusion (SPC; 1,25 *μ*g/g body weight).

### 2.3. Immunohistochemistry

Perfusion-fixed (4% paraformaldehyde), paraffin-embedded sections of SPC-pretreated animals were stained for polymorphonuclear leucocytes (PMN) using the monoclonal antibody MCA771G (Serotec, Oxford, England), developed with antirat peroxidase-coupled secondary antibodies and DAB as a substrate (Vectorstain, DAKO, Germany). TUNEL assays were performed using the ApopTag kit (Chemicon, Temecula, USA). The number of stained cells was semiautomatically determined on three sections per heart using morphometrical analysis software (AnalySIS, Münster, Germany). Apoptosis of rat neonatal cardiomyocytes was induced by exposure to hypoxic conditions (0.8% O_2_ in the medium) for 210 min followed by 150 min of reoxygenation. SPC (10 *μ*M; Sigma, Taufkirchen, Germany) was administered directly before onset of hypoxia. Apoptosis was assessed by TUNEL using the MEBSTAIN Apoptosis Kit II (MBL, Woburn, USA). TUNEL-positive nuclei were counted and expressed as TUNEL-positive/total nuclei.

### 2.4. Flow Chamber Studies

*In vitro* effects of SPC on endothelial adhesiveness for mouse PMNs was determined using a parallel-plate flow chamber model as described in detail previously [7]. PMNs were isolated from bone marrow of mice [13] and labeled using cell tracker green (Molecular Probes, Leiden, Netherlands) before being perfused at 100 s^−1^ across TNF*α*-activated immortalized murine endothelioma cells (fEnd.5). The number of cells with firm adhesion was determined on pictures taken from 15 high-power fields after 5 minutes of cell perfusion followed by 5 minutes of buffer wash and captured on an UltraView (Perkin Elmer, Jügesheim, Germany) confocal scanning microscope. Quantification was performed using Image J software.

### 2.5. Statistical Analysis

Data are presented as mean ± SEM. Nonparametric Kruskal-Wallis testing followed by Dunnett's test was employed to identify significant differences between groups. Significant differences were assumed at *P* < .05 (InStat, GraphPad Inc., San Diego, USA).

## 3. Results

### 3.1. Sphingosylphosphorylcholine (SPC) Reduces Infarct Size after Myocardial Ischemia and Reperfusion In Vivo

**Figure 1:**
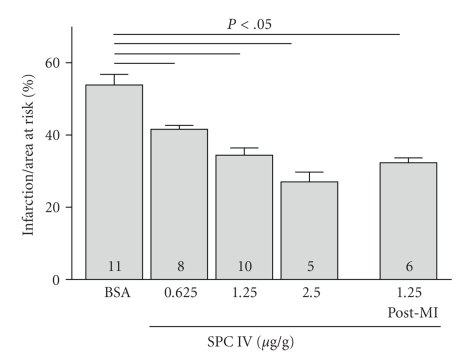
SPC protects against myocardial ischemia/reperfusion injury *in vivo*. SPC (0.625, 1.25 and 2.5 *μ*g/g body weight) and 1% bovine serum albumin in PBS were injected intravenously 30 minutes before and after myocardial ischemia with reinstitution of reperfusion. Infarct size, after MI/R as a function of area at risk is reduced in SPC treated mice.

In wild-type mice, left ventricular cross-sectional area was 13.9 ± 0.7 mm^2^. Ligation of the LAD resulted in an ischemic area of 7.6 ± 0.5 mm^2^ (*n* = 11) constituting the area at risk. The infarcted area measured 3.4 ± 0.4 mm^2^ (*n* = 11). Neither left ventricular area nor area at risk were statistically different between treatment groups. The mortality after myocardial ischemia with reperfusion was about 15% while administration of vehicle control or SPC showed no influence on the rate of mortality in the different treatment groups. In previous work, we showed that S1P 19 and 38 ng/g body weight does-dependently reduced infarct size [7]. Since the K_d_ of S1P receptors for SPC is up to 40-fold higher than that for S1P [14, 15], we administered SPC in an equipotent dose range (0.625, 1.25, and 2.5 *μ*g/g body weight) 30 min before transient coronary artery ligation. Intravenous injection of SPC resulted in a dose-dependent reduction of infarct size by 23%, 36%, and 50%, respectively (*n* = 8,10,5, *P* < .05; Figure 1). Interestingly, when administered therapeutically with reinstitution of reperfusion after myocardial ischemia we also observed a reduction of infarct size by 40% for SPC (1.25 *μ*g/g bw) compared to BSA treated controls (*n* = 6, *P* < .05; Figure 1).

### 3.2. SPC Reduces Leukocyte Recruitment In Vitro and In Vivo

**Figure 2:**
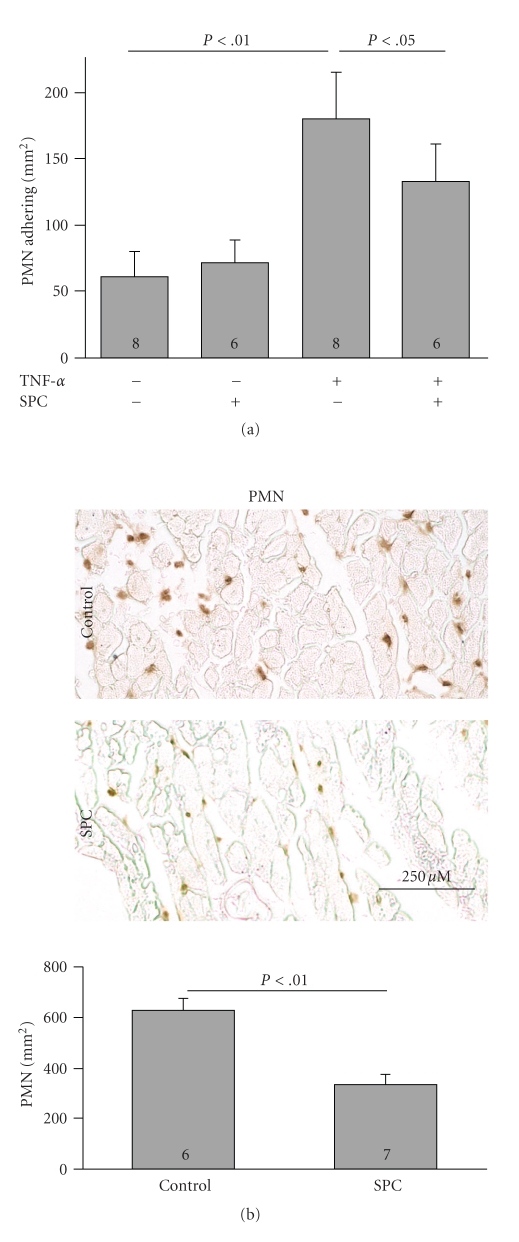
SPC inhibits PMN adhesion to activated endothelium under flow *in vitro* and PMN recruitment in the infarction area *in vivo*. (a) PMN adhesion to TNF-*α*-activated endothelial cells *in vitro* in the presence and absence of 10 *μ*M SPC as quantified in a parallel-plate flow-chamber system. (b) Representative immunohistochemistry and morphometric quantification of PMN in infarcts of vehicle- and SPC-treated mice, respectively, 24 hours after ischemia/reperfusion.

Leukocyte recruitment plays a crucial role in ischemia/reperfusion damage. To test the effect of SPC on leukocyte-endothelial interactions *in vitro*, we used a parallel-plate flow chamber model where mouse macrophages or PMN were perfused over a confluent monolayer of activated murine endothelial cells (fEnd.5), and their adhesion was quantified. Stimulation with TNF*α* increased firm adhesion of PMNs to fEnd.5 by 296 ± 19% (61 ± 19 PMNs/mm^2^ in unstimulated versus 180 ± 35 in TNF*α*-stimulated cells, *n* = 8, *P* < .001). Addition of 10 *μ*M SPC reduced adhesion to 133 ± 28 PMNs/mm^2^ in TNF*α*-stimulated cells (*n* = 6, *P* < .05 versus TNF*α*-stimulated cells in absence of SPC; Figure 2(a)). *In vivo*, during myocardial ischemia/reperfusion, PMN recruitment was decreased from 629 ± 45 PMN/mm^2^ in vehicle-treated hearts to 332 ± 43 PMN/mm^2^ in SPC-pretreated hearts (*n* = 6/7, *P* < .01; Figure 2(b)). The observed antiadhesive effect of SPC *in vitro* does not prove a causal role of SPC on infarct size reduction but suggests that anti-inflammatory effects on endothelial cells may add up to the composite cardioprotective effect *in vivo*.

### 3.3. SPC Protects Cardiomyocytes from Apoptosis In Vitro and In Vivo

**Figure 3:**
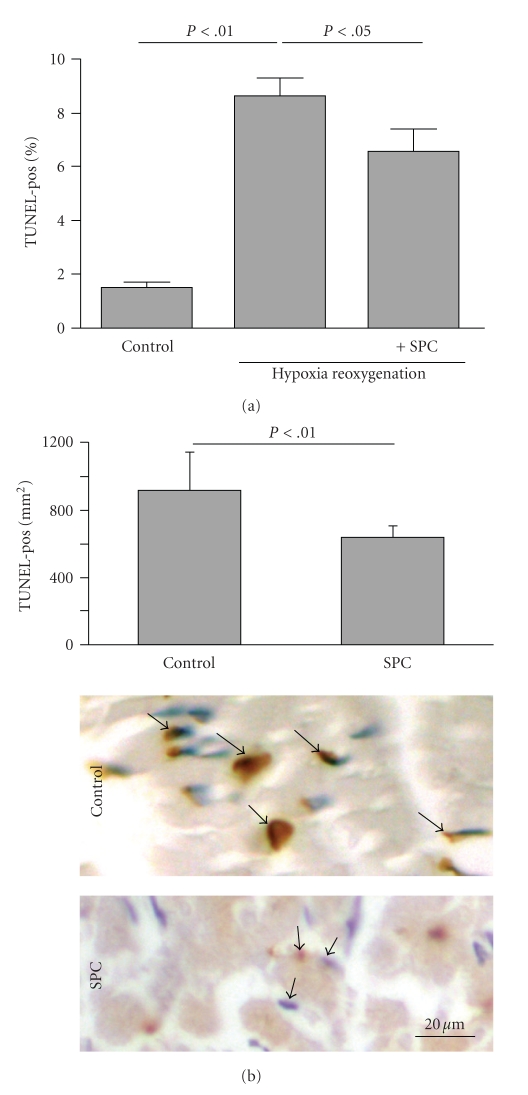
SPC inhibits apoptosis of cardiomyocytes *in vitro* and in the infarction *in vivo*. (a) Quantification of TUNEL-positive nuclei in rat neonatal cardiomyocytes after simulated ischemia/reperfusion in the presence or absence of 10 *μ*M SPC. (b) Representative terminal dUTP nick end-labeling (TUNEL) staining in the area at risk (outside the TTC-positive area) of control and SPC-treated mice 24 hours after ischemia/reperfusion. Morphometric quantification is presented above.

As S1P receptors are present and functional in cardiomyocytes [16] and both HDL and lysophospholipids are potent antiapoptotic signaling mediators in a number of experimental systems [7, 17, 18], we tested if SPC directly protects cardiomyocytes against apoptosis *in vitro*. SPC had an antiapoptotic effect as it significantly reduced the amount of TUNEL-positive nuclei after simulated ischemia/reperfusion (8.7 ± 0.6% versus 6.5 ± 0.9% TUNEL-positive nuclei in control versus SPC treated cardiomyocytes, *n* = 3, *P* < .05; Figure 3(a)). *In vivo*, apoptotic cell death was assessed in mice treated with SPC prior to ischemia. TUNEL-staining in the area at risk (outside the TTC-positive area) was substantially reduced in lysophospholipid-treated mice (920 ± 225 versus 643 ± 66 TUNEL-positive cells/mm^2^ in BSA versus SPC-treated mice, resp.; *n* = 5, *P* < .01; Figure 3(b)). By calculating the amount of apoptotic nuclei per total nuclei in the area at risk (outside the TTC-positive area) we estimated the amount of viable cardiac muscle tissue lost due to apoptosis to be about 17% of the area at risk.

### 3.4. Cardioprotective SPC Effect Is Mediated by the S1P_3_ Lysophospholipid Receptor

**Figure 4:**
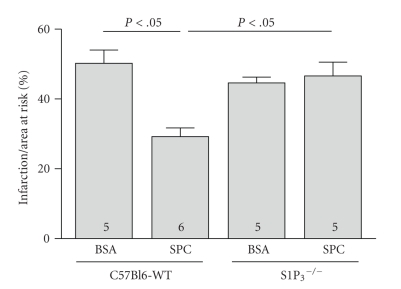
The S1P_3_ lysophospholipid receptor is required for cardioprotection by SPC. Infarct size/area at risk was determined in S1P_3_-deficient mice and their matching wild-type controls (C57BL/6) after treatment with SPC (1.25 *μ*g/g body weight).

In order to investigate which S1P receptor mediates the cardioprotection of SPC, we analyzed the effects of SPC in knockout-S1P_3_ receptor mice (S1P_3_^−/−^) that were available only on the C57BL/6 background. Whereas the studies reported above were carried out in an outbred Swiss strain, the cardioprotection of SPC (1,25 *μ*g/g bw) is present to the same extent in wild-type C57BL/6 mice (29 ± 3,8% versus 34 ± 2% infarction/area at risk, C57BL/6 versus Swiss, *n* = 6, *P* < .05; Figure 4). However, in S1P_3_^−/−^-mice no protection by SPC on infarct size was detectable (105 ± 9% of vehicle-treated control, *n* = 5, *P* = ns; Figure 4).

## 4. Discussion

The salient findings of this study are that HDL-associated SPC, like S1P, exerts cardioprotective antiapoptotic and anti-inflammatory effects when administered preventively prior to ischemia or therapeutically to ischemic myocardium during reperfusion. This effect is mediated through the S1P_3_ receptor and according to our previously published results likely to be nitric oxide dependent. 

Long-term beneficial atheroprotective effects of HDL are generally accepted. Increasing evidence points to additional effects of HDL in connection with acute tissue ischemia independent of its role as cholesterol acceptor. A recent study demonstrated improved functional postischemic recovery of isolated rat hearts by HDL that was attributed to scavenging of myocardially released TNF*α* by HDL [19]. Former studies demonstrated a reduced leukocyte-endothelial interaction in connection with atheroprotection *in vitro* [20] and *in vivo * [21]. 

Rapid reperfusion is an established priority for treatment of myocardial ischemia. The underlying intention is to minimize tissue destruction and thereby infarct size with subsequent improved outcome of the patient. We have shown that HDL, in addition to its effects on reverse cholesterol transport, stimulates NO release in human endothelial cells and induces vasodilatation [2, 10]. According to the aim of rapid reperfusion of an occluded vessel, this may account for direct beneficial effects of HDL on ischemic myocardium. Scar size is, however, not only dependent on tissue loss during ischemia, but increases due to the inflammatory response during reperfusion [22]. Studies with isolated perfused hearts argue against a significant neutrophil-dependent component in cardioprotection, since postconditioning reduced infarct size and necrosis in such leukocyte-free models [23]. However, studies from our group showed that three hours after ischemia, only a small part of the tissue defect is due to leukocytes whereas 24 hours after reperfusion tissue loss is almost twice as big in controls compared to leukocyte depleted animals [7], indicating that neutrophils contribute importantly to a second wave of myocardial tissue loss during later phases. We can not, however, exclude some degree of interdependence of inflammation and apoptosis. That is, inflammatory cell recruitment may contribute to cardiomyocyte apoptosis. One argument supporting this notion is the earlier observation that antileukocyte strategies can entirely prevent tissue damage occurring during later phases of reperfusion [7].

We have recently shown that HDL reduces cardiomyocyte apoptosis and leukocyte recruitment to the postischemic myocardium resulting in a cardioprotective effect. This effect was mediated by HDL's constituent sphingosine-1-phosphate that acts through its receptor S1P_3_. The S1P_3_ effect, in turn, depends on nitric oxide synthase activity [7]. In addition to S1P, sphingosylphosphorylcholine (SPC) is another sphingophospholipid traveling with HDL, and because of their diverse affinities to different receptor subsets, there is an ongoing debate whether SPC and S1P would exert similar or antagonistic effects in the cardiovascular system [2, 24–26]. Furthermore, the distinct role of SPC in different cell types might be diverse. Nixon et al. [27] showed that SPC administered to vascular smooth muscle cells acts as a proinflammatory mediator. In contrast, we here show an anti-inflammatory role of SPC in endothelial cells, suggesting that the balance between SPC effects in different cell types might be an important factor deciding if beneficial or adverse effects are realized in the cardiovascular system. 

Multiple protein kinase and/or phosphatase-signaling pathways are activated during ischemia with reperfusion [23]. Effects of SPC and S1P on downstream kinase phosphorylation have been reported to be divergent in vascular smooth muscle cells from rat cerebral arteries [28]. Therefore, we analyzed the phosphorylation of ERK1/2 and p38MAPK in postischemic tissue and remote myocardium in SPC- versus BSA-treated mice, but we did not observe any significant differences (data not shown). This finding suggests that other signaling pathways might be involved in the *in vivo* function of SPC.

We here demonstrate that the S1P_3_ lysophospholipid receptor is required for cardioprotection by SPC, which is somewhat surprising because SPC is known to have only a low affinity for S1P-receptors [14, 15]. Intracellular and extracellular sphingosine kinases 1 and 2 convert SPC to S1P, which could explain biological similarities of SPC and S1P. While ischemia induces the formation of ceramide and sphingosine by activation of sphingomyelinase, which have been shown to reveal negative effects on cardiac function, it is likely that a rapid and effective conversion of sphingosylphosphorylcholine to S1P catalyzed by sphingosine kinase [24] might be the underlying effect of cardioprotection by SPC. 

Activation of sphingosine kinase (SphK) has been shown to play a crucial role in protection against apoptosis in oligodendrocyte survival by neutrophin-3 [29]. Furthermore, Jin and Karliner [30] reported cardioprotection via a PKCepsilon-SphK-S1P-Akt pathway.

We can not exclude that SPC pretreatments sets of cascades are also involved in preconditioning phenomena, especially since nitric oxide seems to be one of the active motifs. The postischemic treatment effects that we observed do not likewise exclude that postconditioning effects are accountable. Nevertheless, there is an antiapoptotic and anti-inflammatory effect involved in SPC-fostered cardioprotection.

## 5. Conclusion

In aggregate, our data suggest that SPC, like S1P, exerts cardioprotective effects during reperfusion injury regardless of the timing of its administration. Even if HDL rising strategies would, in parallel, increase circulating bioactive S1P along with SPC, no adverse effects of SPC will antagonize S1Ps beneficial effects. The perspectives of interventions designed to acutely raise HDL levels in patients at high risk, for example, such with acute coronary syndromes to improve prognosis may be very attractive both for patients and clinicians.
